# Determinant factors of the longitudinal pulse pressure among hypertensive patients treated at Assosa general hospital, Western Ethiopia

**DOI:** 10.1186/s12872-024-04389-7

**Published:** 2024-12-19

**Authors:** Maru Zewdu Kassie, Haymanot Berelie Berehan, Seyifemickael Amare Yilema, Berhanie Addis Ayele

**Affiliations:** 1 Assosa University, Assosa, Ethiopia; 2 Debre Tabor University, Debre Tabor, Ethiopia; 3 Gondar University, Gondar, Ethiopia

**Keywords:** Pulse pressure, Cardiovascular disease, Hypertension, Linear mixed model, Longitudinal data

## Abstract

**Background:**

Hypertension is a common, long-term condition that tends to be associated with age and can lead to significant cardiovascular complications. This study aimed to identify factors influencing the longitudinal Pulse Pressure of hypertensive patients treated at Assosa General Hospital (AGH), Western Ethiopia.

**Methods:**

A retrospective study design was conducted from 325 randomly selected HTN patients in the outpatient department (OPD) clinic at AGH during the follow-up period from January 2022 to January 2024. The analysis included exploratory data analysis and the application of a linear mixed model. This model was used to analyze the longitudinally measured pulse pressure in patients with hypertension. The appropriate variance-covariance structure chosen for this analysis was the unstructured (UN) format.

**Result:**

Among the 325 patients included in the study, 51.5% were female, and 54.2% were from urban areas. The variables: Age (p-value < 0.0001), Urban (p-value = 0.012), FHHTN (p-value < 0.0238), Stage-I HTN (p-value = 0.0403), Stage-II HTN (p-value = 0.0022), DM (p-value < 0.0001), CKD (p-value < 0.0001), Smoking (p-value < 0.0001), Enalapril + Nifedipine (p-value = 0.0249), and follow-up time (p-value < 0.0001) were significant factors for the progression of pulse pressure.

**Conclusion:**

The profile plot showed that the patient’s pulse pressure decreases slowly as follow-up time increases. Age, Residence, FHHTN, DM, CKD, Smoking status, and Stages of HTN were positively associated with pulse pressure, whereas Treatment type and follow-up time were negatively associated with pulse pressure. So, Healthcare providers should prioritize addressing the modifiable risk factors mentioned above to help mitigate the progression of blood pressure specifically pulse pressure in hypertensive patients.

**Supplementary Information:**

The online version contains supplementary material available at 10.1186/s12872-024-04389-7.

## Introduction

Cardiovascular disease (CVD) is the major worldwide public health challenge and the principal cause of death, with 17.3 million deaths per year and a projected increase to more than 23 million by 2030 [1, 2]. High blood pressure remains the leading cause of death globally, accounting for 10.4 million deaths per year [3]. The standard definition of Hypertension includes an elevation of Systolic blood pressure (SBP) and diastolic blood pressure (DBP); thus, a person is said to be hypertensive if his/her systolic blood pressure (SBP) is greater than 140 mmHg and/or diastolic blood pressure (DBP) is greater than90 mmHg and those who were already under medication [4, 5]. However, in addition to SBP & DBP; the principal components of BP consist of both a pulsatile component (Pulse Pressure, PP) and a steady component (Mean Arterial Pressure, MAP).MAP is mostly determined by cardiac output and vascular resistance while, PP depends on arterial stiffness, ventricular ejection, and timing of wave reflections [6, 7].

It remains unclear which measures of BP, either alone or in combination; best predicts mortality and other CVD risk. Data from coronary heart disease [6, 8] and other studies [9–11] indicates that SBP steadily increases over all age ranges, while DBP rises until age 60 years and then starts to decline continuously. Thus, the gap between SBP & DBP tends to wide. Consequently, PP (the difference between SBP & DBP) may become a more important BP measure associated with CVD risk in older individuals. Cardiovascular risk (CVR) increased with lower DBP at any level of SBP > 120 mm Hg in middle-aged and elderly, suggesting that higher PP was an important predictor of cardiovascular risk [11]. Another research indicates that the steady component of blood pressure is a strong risk factor for cardiovascular death in both sexes, While the pulsatile component may be an independent risk factor for women over 55 [12]. Another study suggests that systolic pressure is actually the more potent contributor to cardiovascular risk [13, 14]. This all evidence recommends that PP is an important determinant factor of cardiovascular events than SBP & DBP mostly in older patients. But, these evidences were done by using one time (crossectional data), and also the impact of PP on CVD risk was high mostly in older patients [8]. Therefore, the authors initiated this study to examine whether the above evidence fits and to assess the impact of longitudinal PP measures on CVD risk.

Identifying the determinant predictors of pulse pressure is crucial for controlling blood pressure and minimizing the risk of cardiovascular complications. Several studies have been conducted on pulse pressure in the past. A long-term follow-up study (23 years) suggested that several well-known cardiovascular risk factors, including glucose levels, BMI, heart rate, family history of hypertension, and particularly cholesterol, are predictors of increased pulse pressure in both genders [15]. Another study conducted on chronic hemodialysis patients showed that an increase in pulse pressure was positively associated with increased age, the presence of diabetes mellitus, interdialytic weight gain, and current smoking, while it was negatively associated with hemoglobin concentration [16]. Additionally, the study indicated that central pulse pressure (PP) is influenced by total arterial compliance and ventricular dynamics [17–19].

Furthermore, another study concluded that an increasing metabolic syndrome score is an independent determinant of increased pulse pressure and arterial stiffness [20]. Also, the study [21], concluded that an increasing metabolic syndrome score is an independent determinant of increased pulse pressure and arterial stiffness. According to additional studies, pulse pressure a marker of arterial stiffening is suggested to be an independent determinant of the treatment-associated decline in renal function in essential hypertension [21–24]. Elevated blood pressure is known to be a risk factor for the development of several diseases [25, 26]. Its progression is strongly associated with cardiac and vascular abnormalities, leading to cardiovascular complications such as renal impairment, kidney disease, coronary heart disease, stroke, heart failure, and dementia [11, 27]. Therefore, controlling blood pressure is essential for maintaining health and preventing cardiovascular complications.

Even if the prevalence of hypertension is substantially increasing in Ethiopia as well as in the study area and also even if there are many studies conducted for hypertension and related cardiovascular disease, as far as the investigator knowledge is concerned, there is scarce of a study conducted on the determinants of longitudinal pulse pressure for hypertensive patients in the study area. So, this study focused on identifying factors that influence the longitudinal Pulse Pressure of hypertensive patients treated at Assosa General Hospital, Ethiopia.

## Methods

### Description of study area and design

The study was conducted at Assosa General Hospital, Assosa, Western Ethiopia. The area is located 670 km far from Addis Ababa, the capital city of the country. The hospital has specialty chronic illness clinics where patients with specific chronic diseases are referred for follow-up. A retrospective study design was carried out to retrieve relevant information from the medical records of HTN patients to address the objective of the study.

### Source of data and data collection procedures

HTN patients were a source of data for this study. The data was collected from the medical chart of HTN patients in the OPD (outpatient department) section at the hospital who were under follow-up from January 2022 to January 2024. The data were collected by three statisticians and one nurse recruited from April 15, 2024, to May 03, 2024. The longitudinal data was extracted from the secondary data seated at the patient’s chart which contains socio-demographic and clinical information of HTN patients under the follow-up. The longitudinal outcome variable PP is measured approximately every 3 months irrespective of patient visits to the OPD section of chronic disease at AGH. The patient charts are prepared by the Federal Ministry of Health for uniform use by clinicians to identify and document clinical and laboratory measurements early. Thus, this study used secondary data obtained from patient follow-up charts, and there was no need to involve study participants.

### Inclusion and exclusion criteria

All newly diagnosed HTN patients at AGH from January 2022 to January 2024, as well as patients who have at least two follow-ups, were included in the study. Conversely, those who develop CVD complications at the start of the follow-up, who start medication before January 2022, and who have less than two follow-ups were excluded. Here there were 745 HTN patients in the study period. Of these, only 325 of them satisfy these inclusion criteria. Therefore, these 325 patients were followed.

### Operational definitions

#### Follow-up time

The specific time intervals at which a patient returns to the healthcare facility for subsequent visits to monitor their health status and treatment progress.

#### Medication type

The specific category or class of medication prescribed to a patient for the treatment of hypertension.

#### Enalapril

An ACE inhibitor used to treat high blood pressure and heart failure by relaxing blood vessels.

#### Nifedipine

A calcium channel blocker that lowers blood pressure and treats angina by relaxing blood vessels.

**Pulse Pressure**, the difference between SBP and DBP, is a key marker for cardiovascular health, reflecting arterial stiffness and cardiovascular event risk.

#### Target blood pressure levels

The target blood pressure levels followed in this study were based on the recommendations from the Ethiopian Ministry of Health and WHO. The target for SBP was < 120 mmHg, and for DBP was < 80 mmHg.

## Variables in the study

### Response variables

The response variable in the current investigation was pulse pressure, which is calculated as $\:\text{P}\text{P}=\text{S}\text{B}\text{P}-\text{D}\text{B}\text{P}$, where SBP and DBP represent systolic blood pressure and diastolic blood pressure, respectively.

The longitudinal outcome variables, SBP and DBP, are measured approximately every 3 months, regardless of patient visits to the OPD section of chronic disease at AGH. These measurements are taken at the start of treatment, as well as at the 3-, 6-, 9-, 12-, 15-, 18-, 21-, and 24-month visits (i.e., *n* = 9). We have assigned follow-up time as (1, 2, 3, 4, 5, 6, 7, 8, and 9) visits.

### Independent variables

The study considers the following potential explanatory variables: Age in years, Sex (male, female), Place of residence (rural, urban), Presence of Diabetes disease (no, yes), Presence of TB (no, yes), Presence CKD (no, yes), Smoking Status (no, yes), Family history of HTN (no, yes), Stages of HTN (Pre-stage, I and II), and Medication type (Enalapril, Nifedipine, Enalapril + Nifedipine, others).

### Data Analysis

The data was analyzed using R-4.22 and SAS 9.4. Descriptive statistics such as frequency tables and percentages for baseline categorical covariates were used to summarize the distribution of selected background characteristics of the sample. Also, normal Q-Q plot and profile plots were used to explore the data as well as to check the normality and linearity assumptions of the data.

### Longitudinal data analysis

Measurements made on the same variable for the same subject are more likely to be correlated, models fitted to longitudinal or repeated measures data involve the estimation of covariance parameters to capture this correlation [28]. In this case standard statistical methods like simple linear regression that assume independent observations are not appropriate. Thus, in this study linear mixed-effects model was used for the analysis of continuous longitudinal response.

One of the major objectives of statistical analysis is to address variations in the data. There are two sources of variations considered in the longitudinal data sets. Those are the within-subject and between-subject variations. The former helps us to study changes over time, and the latter helps us to understand differences between subjects. To deal with longitudinal data with continuous outcomes, the widely used method is the linear mixed effects model [29].

A linear mixed model was fitted to estimate the effect of each demographic and clinical factor on the progression of pulse pressure. The Linear Mixed Effect Model is a model that contains both fixed and random effects. Thus, the fixed effect part of the model represents the mean response, while the random effect part represents the individual-level responses. Let $\:{y}_{i1},\:\:{y}_{i2},\:\:\dots\:,\:\:{y}_{in}$ is the measurement for blood sugar measured at time $\:{\:t}_{i1},\:\:{t}_{i2},\:\:\dots\:,\:\:{t}_{in}$; the linear mixed model of the data which is proposed by Laird and ware is expressed as [29].

3.1 $${y_i} = {X^\prime }\left( t \right)\beta + {Z^\prime }_i\left( t \right){b_i} + {\varepsilon _i}$$

i.e.$$\>\>\>\>\>\>\>\>\>\>\>\>\>\>\>\>\>\>\>\>\>y{\>_i} = \mu {\>_i}\left( t \right) + {U_{1i}}\left( t \right) + {\varepsilon _i}$$

Where $\:{y}_{i}$ is the $\:nx1$ vector of observed response values, β is the $\:p\:x\:1$vector of fixed effect parameter, $\:x\left(t\right)$ is the $\:{n}_{i}x\:p$ observed design matrix of corresponding to the fixed effect, $\:{b}_{i}$ the $\:qx1$ vector random effect parameter, $\:{z}_{i}$ is the $\:{n}_{i}xq$ observed design matrix corresponds to the random effect, and ${\varepsilon _i}$ is the $\:{n}_{i}x\:1$ vector of residual for the response. The corresponding assumption for the model (3.1) is${b_i} \sim N(0,D)$ and ${\varepsilon _i}\sim$N (0, Σ), where D and Σ are the variance-covariance matrix for $\:{b}_{i}$and ${\varepsilon _i}$ for the outcome variable respectively.

### Covariance structure

In this study, we considered three covariance structures: Compound Symmetry (CS), First-Order Autoregressive (AR(1)), and Unstructured (UN) to model the repeated measures of pulse pressure in hypertensive patients.

The **Compound Symmetry (CS)** structure assumes a constant correlation between repeated measures at all-time points, regardless of their temporal spacing. While this structure simplifies the model, it may not adequately capture the evolving dynamics of pulse pressure in clinical settings, where correlations between measures typically decrease as the time between measurements increases [28, 29].

The corresponding correlation matrix is:

$$\Sigma = \left[ {\matrix{{\sigma {\>^2}} & {\>\sigma {\>^2}\rho \>} & {\sigma {\>^2}\rho } & \ldots & {\sigma {\>^2}\rho \>} \cr {\sigma {\>^2}\rho \>} & {\sigma {\>^2}} & {\sigma {\>^2}\rho \>} & \cdots & {\sigma {\>^2}\rho \>} \cr {\sigma {\>^2}\rho \>} & {\sigma {\>^2}\rho \>} & {\sigma {\>^2}} & \cdots & {\sigma {\>^2}\rho \>} \cr \vdots & \vdots & \vdots & \ddots & \vdots \cr {\sigma {\>^2}\rho \>} & {\sigma {\>^2}\rho \>} & {\sigma {\>^2}\rho \>} & \cdots & {\sigma {\>^2}} \cr } } \right]$$

The **Autoregressive order one (AR** [1]**)** covariance structure is a special case of the Toeplitz covariance structure and is useful for modeling first-order temporal autocorrelation. The AR(1) structure is typically used to fit models for equally spaced longitudinal observations on the same units of analysis. In this structure, observations closer in time exhibit higher correlations than those farther apart.

The general form of the Σ matrix for this covariance structure is:

$$\Sigma = \left[ {\matrix{{\sigma {\>^2}} & {\sigma {\>^2}\rho } & {\sigma {\>^2}\rho {\>^2}} & \cdots & {\sigma {\>^2}\rho {\>^{n - 1}}} \cr {\sigma {\>^2}\rho } & {\sigma {\>^2}} & {\sigma {\>^2}\rho } & \cdots & {\sigma {\>^2}\rho {\>^{n - 2}}} \cr {\sigma {\>^2}\rho {\>^2}} & {\sigma {\>^2}\rho } & {\sigma {\>^2}} & \cdots & {\sigma {\>^2}\rho {\>^{n - 3}}} \cr \vdots & \vdots & \vdots & \ddots & \vdots \cr {\sigma {\>^2}\rho {\>^{n - 1}}} & {\sigma {\>^2}\rho {\>^{n - 2}}} & {\sigma {\>^2}\rho {\>^{n - 3}}} & \cdots & {\sigma {\>^2}} \cr } } \right]$$

The **Unstructured (UN)** covariance structure is one of the most flexible models for analyzing repeated measures or longitudinal data. It does not impose any specific mathematical pattern or constraints on the relationships (correlations and variances) between repeated measurements within the same subject or experimental unit. Instead, it estimates a unique variance for each time point and a unique covariance for each pair of time points. This flexibility comes at the cost of increased complexity, as a $\:pxp$ covariance matrix has$\:\:\:\frac{p(p+1)}{2}$ non-redundant elements to estimate.

The general form of the Σ matrix for this covariance structure is:

$$\Sigma = \left[ {\matrix{{\sigma {\>^2}_1} & {\sigma {\>_{12}}} & {\sigma {\>_{13}}} & \cdots & {\sigma {\>_{1p}}} \cr {\sigma {\>_{21}}} & {\sigma {\>^2}_2} & {\sigma {\>_{23}}} & \cdots & {\sigma {\>_{2p}}} \cr {\sigma {\>_{31}}} & {\sigma {\>_{32}}} & {\sigma {\>^2}_3} & \cdots & {\sigma {\>_{3p}}} \cr \vdots & \vdots & \vdots & \ddots & \vdots \cr {\sigma {\>_{p1}}} & {\sigma {\>_{p2}}} & {\sigma {\>_{p3}}} & \cdots & {\sigma {\>^2}_p} \cr } } \right]$$

The UN covariance structure is particularly relevant for longitudinal data where variance and correlation may vary significantly over time. This is often observed in clinical contexts where factors such as disease progression, treatment effects, or other time-varying covariates influence measurements.

Furthermore, selecting the appropriate covariance structure is critical and should be based on certain criteria such as the Akaike Information Criterion (AIC) and Bayesian Information Criterion (BIC), which indicate a better overall model fit.

### Random effects model

In this study, random effects models were employed to analyze the repeated measures of pulse pressure in hypertensive patients. These models account for both the within-subject correlation of repeated observations and the between-subject variability. Specifically, two types of random effects models were used: the random intercept model and the random intercept and slope model [28, 29].

### Random intercept model

The random intercept model allows the intercepts to vary across individuals, capturing the between-subject variability in baseline pulse pressure. This model consists of two distinct components:

1. **Fixed Effects**: Represented by the population-level average, consisting of the intercept and the coefficients of explanatory variables multiplied by their respective covariates.
2. **Random Effects**: Captures the individual-specific deviations from the population average. The model assumes:

- ${\varepsilon _i} \sim N\left( {0,{\sigma ^2}} \right)$, the within-subject error term.
- $\:{\:b}_{i}\sim\:N(0,{{\sigma\:}^{2}}_{b})$, the random intercept effect for individual $\:i$.
- Independence assumptions:

$\:Cov({b}_{i};\:{b}_{j})\:=\:0\:if\:i\:\ne\:\:j$, Cov (${\varepsilon _i},{\varepsilon _j}$) = 0 if i $\:\ne\:$ j, and Cov($\:{b}_{i}$; ${\varepsilon _i}$) = 0

The random intercept model is expressed as:

3.2 $${y_{ij}} = \>\beta {\>_0} + \beta {\>_1}{x_{ij}} + {b_{0i}} + {\varepsilon _{ij}}$$

Where $\:{\:y}_{ij}$represents the pulse pressure for individual $\:i$ at time $\:j$, $\:{\beta\:}_{0}$ is the population level intercept, $\:{\beta\:}_{1}$ is the fixed effect of covariate $\:{x}_{ij}$, $\:{b}_{0i}$ is the random intercept for individual $\:i$, and ${\varepsilon _{ij}}$ is the within-subject error term.

### Random intercept and slope model

This model extends the random intercept model by allowing both the intercept and slope to vary across individuals. It captures the variability in how individuals respond differently to covariates over time.

The model is expressed as:

3.3 $${y_{ij}} = \>\beta {\>_0} + \beta {\>_1}{x_{ij}} + {b_{0i}} + {b_{1i}}{z_{ij}} + {\varepsilon _{ij}}$$

Where $\:{b}_{1i}$ represents the random slope effect of the covariate $\:{z}_{ij}$. In this case, two additional parameters are estimated:

1. The variance of the random intercepts $\:{{\sigma\:}^{2}}_{b0}$
2. The variance of the random slopes $\:{{\sigma\:}^{2}}_{b1}$

The structure of the random effects is defined as:

$$\left( {\matrix{{\beta {\>_0}} \cr {\>\beta {\>_1}} \cr } } \right) \sim N\left( {0,{D_i}} \right)\>with\>{D_i} = \left[ {\matrix{{\sigma {\>^2}_{b0}} & {\>\sigma {\>_{b0b1}}} \cr {\>\sigma {\>_{b0b1}}} & {\>\sigma {\>^2}_{b1}} \cr } } \right]$$

Where $\:{\sigma\:}_{b0b1}$ denotes the covariance between the intercepts and slopes.

The goodness of fit test was checked using BIC, AIC, and LRT [30, 31].

## Result

The summary statistics of predictor variables in the data were displayed in Table 1. Among the 325 patients included in the study, 167(51.5%) were females and the rest 158(48.5%) were males, and 54.2% were from urban areas. About 48(14.8%) had FHHTN and 108(33.2%) had DM in addition to HTN. Regarding to clinical stage of HTN 106(32.7%), 134(41.1%) and 85(26.2%) were pre-stage, stage I, and II respectively. Regarding to medication type 110(33.7%), 98(30.2%), 91(27.9%), and 26(8.2%) of HTN patients used Enalapril, Nifedipine, Enalapril + Nifedipine and others respectively.

The baseline measured continuous covariates include age, baseline lnPP (logarithm of pulse pressure), baseline SBP (systolic blood pressure), and baseline DBP (diastolic blood pressure). The mean age of participants was 47.2 years (SD = 5.7), reflecting a relatively consistent age distribution, which is an important factor in the progression of hypertension and related comorbidities. The baseline lnPP, calculated as the natural logarithm of pulse pressure (the difference between SBP and DBP), had a mean of 3.946 (SD = 0.087), indicating minimal variation in pulse pressure across participants. The mean baseline SBP was 142 mmHg (SD = 9.6), suggesting variability in systolic pressure levels. The baseline DBP had a mean of 98 mmHg (SD = 6.0), representing the typical diastolic pressure within the study population.

**Table 1:** Summary statistics for the variables included in the study

Variable	Category	Total	Percentage	
Sex	Female	167	51.5	
	Male	158	48.5	
Residence	Rural	149	45.8	
	Urban	176	54.2	
FHHTN	No	277	85.2	
	Yes	48	14.8	
Stage of HTN	Pre-stage	106	32.7	
	Stage I	134	41.1	
	Stage II	85	26.2	
DM	No	217	66.8	
	Yes	108	33.2	
CKD	No	260	80.1	
	Yes	65	19.9	
TB	No	248	76.3	
	Yes	77	23.7	
Smoking	No	296	91.3	
	Yes	29	8.7	
Medication type	Enalapril (ref)	110	33.7	
	Nifedipine	98	30.2	
	Enalapril + Nifedipine	91	27.9	
	Others	26	8.2	
**Baseline measured continuous covariates**				
			**Mean**	**SD**
Age in years		325	47.184	5.715
Baseline lnPP in mmHg		325	3.946	0.087
Baseline SBP in mmHg		325	142	9.649
Baseline DBP in mmHg		325	98	6.037

### Data exploration for longitudinal data

Exploratory data analysis was conducted in order to investigate various associations, structures and patterns exhibited in the data set. In addition, the individual profile plots and mean structure plots were obtained in order to gain some insights into the data.

### Individual profile plot

An Individual Profile Plot is a type of visualization used to track how a particular variable changes for each individual over time. It is especially useful in longitudinal data analysis, where repeated measurements are collected from the same subjects at different time points. In this plot, each line represents an individual’s trajectory, with the x-axis typically indicating time (e.g., months or weeks) and the y-axis representing the variable of interest (e.g., pulse pressure or blood pressure).

**Fig. 1:**
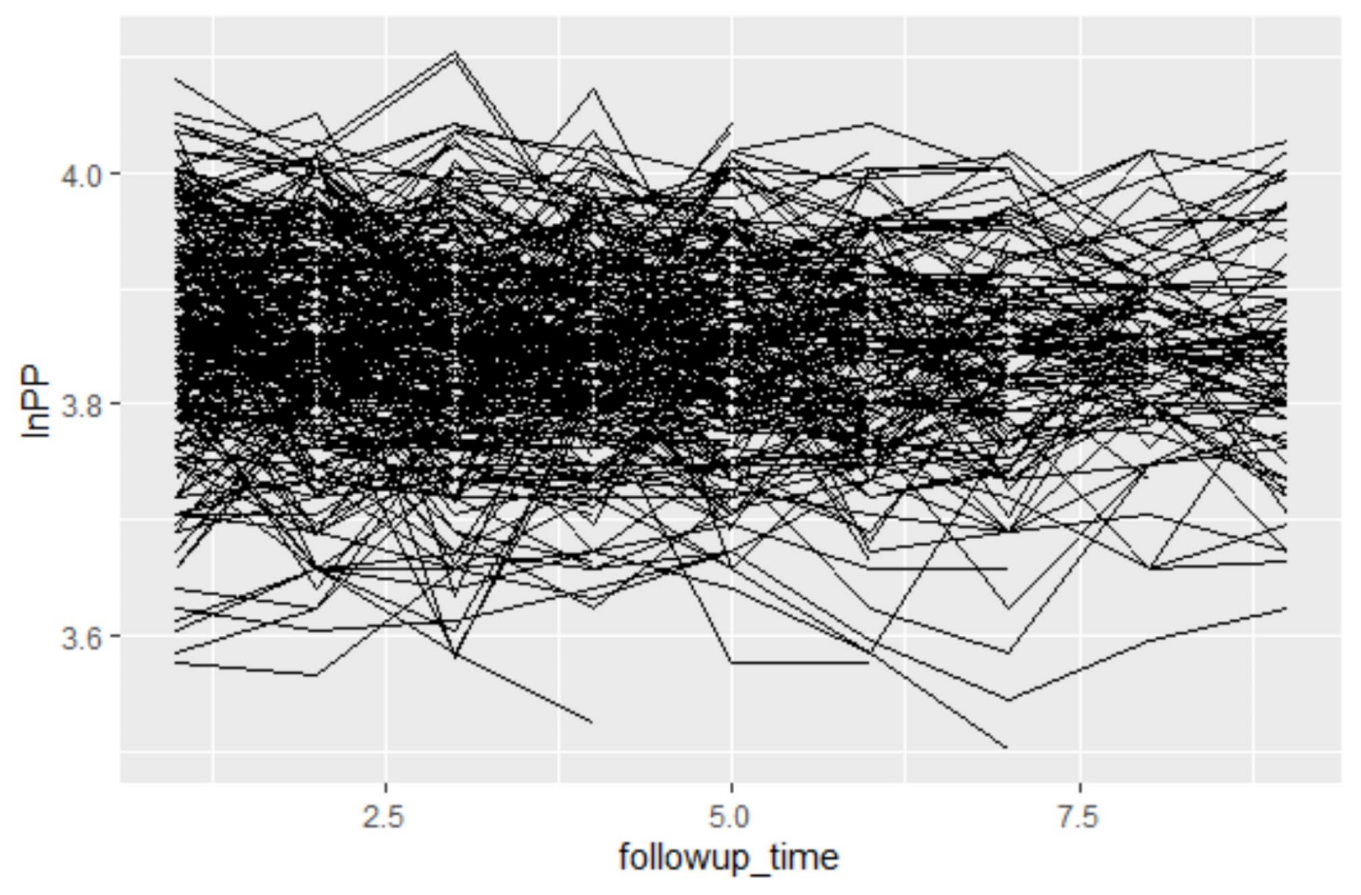
Individual Profile plot for HTN patients

In this study, the Individual Profile Plot depicted the pattern of change in pulse pressure (PP) among hypertensive patients over time. The plot showed a gradual reduction in PP throughout the follow-up period, highlighting the linear effect of time on the progression of hypertension. This steady decline suggests that targeted interventions and antihypertensive therapy are effective in managing the condition. Figure 1 further illustrated this decreasing trend in PP over time, emphasizing the importance of understanding the progression rate of hypertension in terms of PP to optimize treatment strategies. The observed reduction in PP underscores the positive impact of timely and targeted medical interventions.

### Loess smoothing plot

A Loess Smoothing Plot is a statistical visualization used to identify and display trends in data by fitting a locally weighted regression curve to the points in the dataset. This type of plot is particularly useful for analyzing the overall patterns in a variable across a continuous predictor, such as time, without assuming a strict linear relationship. The smoothed curve represents the average trend, effectively reducing the noise caused by individual variations.

**Fig. 2:**
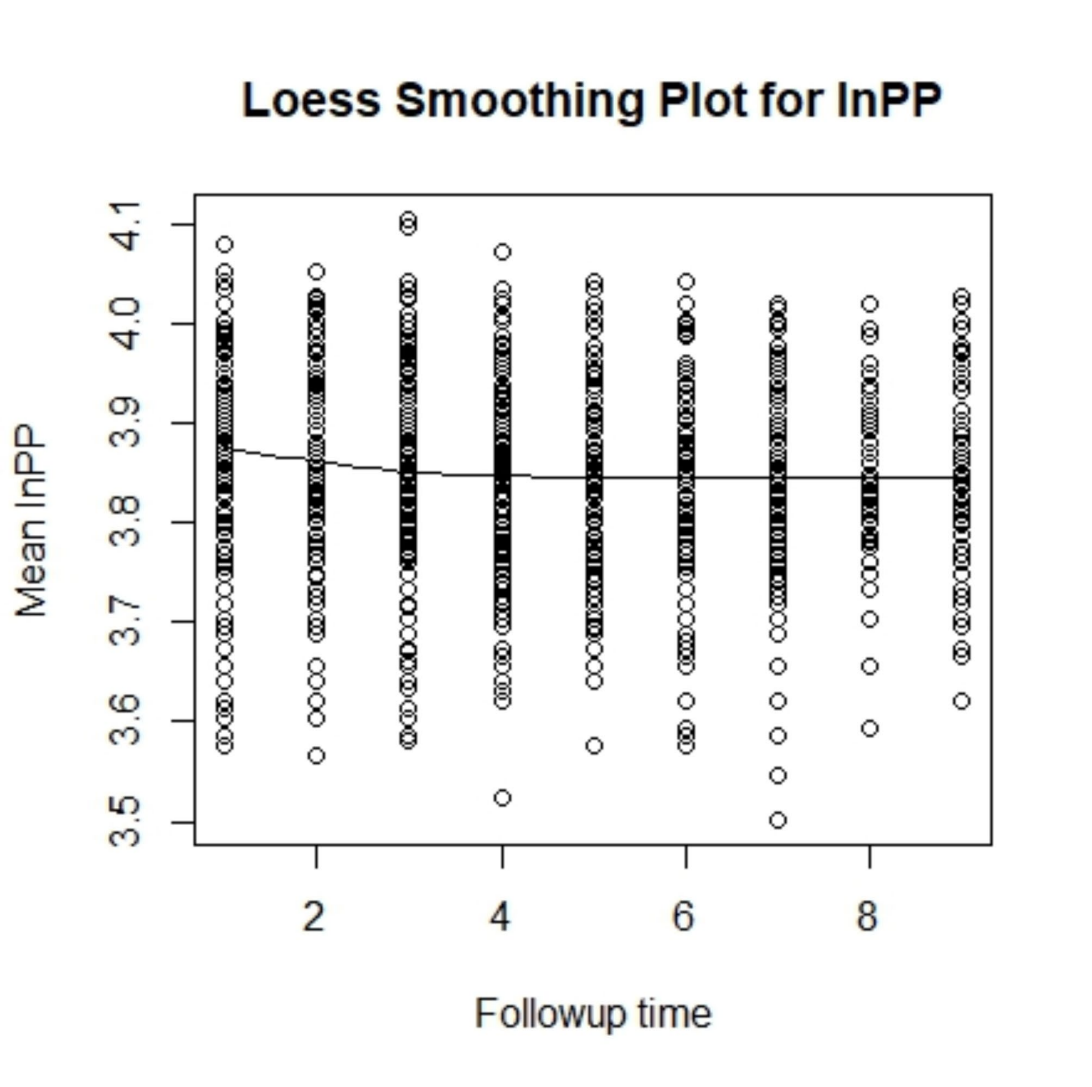
Loess Smoothing Plot for the log-transformed pulse pressure

In this study, the Loess Smoothing Plot visualizes the mean log-transformed pulse pressure (lnPP) over the follow-up time among hypertensive patients. The x-axis represents the follow-up time, while the y-axis shows the mean lnPP. The smoothed curve illustrates a gradual and consistent decline in lnPP as time progresses, highlighting the general trend of decreasing pulse pressure during the follow-up period. This decline underscores the potential effectiveness of antihypertensive therapies and targeted interventions in controlling blood pressure over time Fig. 2.

In addition to pulse pressure, SuppFugure 1 and SuppFigure 2 demonstrate that the smoothed curves reveal a gradual and consistent decline in SBP and DBP over time. This trend highlights the general pattern of decreasing SBP and DBP during the follow-up period. However, the rate of decrease in DBP is notably higher than that of SBP. Clinically, while understanding the changes in pulse pressure with and without adjustment for SBP is important, adjustments for SBP may not be very critical, as the trends for SBP and pulse pressure were almost identical.

Compared to an Individual Profile Plot, which tracks the trajectories of individual subjects, the Loess Smoothing Plot provides a broader, population-level view of the overall trend, making it an essential tool for understanding the collective progression of hypertension in the study cohort.

### Selection of covariance structure in linear mixed model

After exploratory data analysis, good models that best describe the observed average trends and also reflect the observed correlation structures were sought for the data sets. The three commonly used covariance structures which are compound symmetry (CS), unstructured and first-order autoregressive AR(1) were considered. As we have seen from Table 2 the AIC and BIC values for unstructured covariance structure were smaller than the rest, so unstructured covariance structure was selected due to the smallest AIC and BIC compared to the remaining covariance structures, indicating better model fit. Moreover, the UN structure effectively captures the complex correlations among measurements at different time points, which is critical for understanding the variability in pulse pressure among hypertensive patients. This approach aligns with the clinical understanding that correlations between repeated measures of pulse pressure may vary significantly over time.

**Table 2:** Comparison of covariance structure for linear mixed-effects model

Covariance structure	AIC	BIC	LogLik
AR(1)	-4870.019	-4763.394	2454.009
CS	-4792.907	-4686.282	2415.454
UN	-4794.907	-4693.894	2415.454

### Selection of random effects in linear mixed model

Since the unstructured covariance structure was identified as the best choice for modeling the covariance structure, we implemented various linear mixed models to analyze the longitudinal pulse pressure data, incorporating subject-specific random effects. To determine the most suitable random effects structure, we compared the information criteria values (AIC and BIC) for models with random intercepts, random slopes, and both random intercepts and slopes.

As shown in Table 3, the random intercept and slope model had the lowest AIC and BIC values, indicating the best fit for the data. This model allows both the intercept (baseline pulse pressure) and the slope (rate of change in pulse pressure over time) to vary randomly across individuals.

These findings emphasize the importance of personalized management strategies for hypertensive patients, as both baseline differences and varying responses over time significantly impact clinical outcomes. By including both random intercepts and slopes, the model accounts for this heterogeneity, providing a robust framework for analyzing longitudinal data.

In summary, the random intercept and slope model was selected based on its ability to capture both individual baseline variations and differences in the trajectory of pulse pressure over time, as reflected by its superior model fit criteria (lowest AIC and BIC values).

**Table 3:** Selection of random effects to be included in the linear mixed model

Models for random effects	AIC	BIC	LogLik
Random Intercept	-4794.907	-4693.894	2415.454
Random Slope	-4502.678	-4401.665	2269.339
Random Intercept & Slope	-4835.517	-4723.28	2437.759

### Multivariable analysis for linear mixed model

It was done by all predictor variables significant at a 25% level of significance, as we have seen from Table 4 the variables age, Residence, FHHTN, Stages of HTN, Presence of DM, CKD, Smoking Status, Treatment Type, and Follow-up time in months have significance association with Pulse pressure measurements at 5% level of significance. Also, all the random effect parameters were statistically significant.

The estimated coefficient of fixed effect intercept was 6.7669, which indicates that the average mean value of PP for patients was 6.7669 mmHg keeping the effect of other factors constant (p-value < 0.0001). For a unit increased in age, the average PP of patients was significantly increased by 0.043 mmHg (p-value = 0.0007) keeping all other variables constant.

The average PP of urban patients was significantly higher by 0.0259 mmHg (p-value = 0.0120) compared to rural patients keeping other variables remaining constant. The average PP of the patients with FHHTN was significantly higher by 0.0637mmHg (p-value = 0.0238) compared to the patients with no FHHTN keeping other variables remaining constant. The average PP of Stage-I HTN patients was significantly higher by 0.0859 mmHg (p-value = 0.0403) compared to Pre-stage HTN patients keeping other variables remaining constant. The average PP of Stage-II HTN patients was significantly higher by 0.0935 mmHg (p-value = 0.0022) compared to Pre-stage HTN patients keeping other variables remaining constant. The average PP of patients who had DM was significantly higher by 0.0268 mmHg (p-value < 0.0001) compared to patients who hadn’t DM keeping other variables remaining constant. The average PP of patients who had CKD was significantly higher by 0.0457 mmHg (p-value < 0.0001) compared to patients who hadn’t CKD keeping other variables remaining constant.

The average PP of smoker patients was significantly higher by 0.0682 mmHg (p-value < 0.0001) compared to non-smokers keeping other variables remaining constant. The average PP of patients who had used Nifedipine and Enalapril treatments mutually was significantly lowered by 0.0879 mmHg (p-value < 0.0001) compared to patients who had used other types of treatment keeping other variables remaining constant. For a unit increase in visit time, the average PP of HTN patients was significantly increased by 0.0581 mg/dl (p-value < 0.0001) keeping other variables constant.

**Table 4:** Result of the final linear mixed model for T1DM

Covariate	Estimate	SE	95% CI		*p*-value
			Lower	Upper	
Intercept	3.948	0.0617	3.8270	4.0689	< 0.0001^***^
Age	0.043	0.0128	0.0179	0.0681	0.0007^***^
Sex(ref = Male)					
Female	0.0725	0.0561	-0.0374	0.1824	0.1962
Residence (ref = Rural)					
Urban	0.0259	0.0102	0.0059	0.0458	0.0120^*^
FHHTN (No)					
Yes	0.0637	0.0282	0.0084	0.1189	0.0238^*^
Stages of HTN (Ref = Pre-stage)					
Stage I	0.0859	0.0419	0.0037	0.1680	0.0403
Stage II	0.0935	0.0306	0.0335	0.1534	0.0022
DM (ref = No)					
Yes	0.0268	0.0046	0.0177	0.0358	< 0.0001^***^
CKD (ref = No)					
Yes	0.0457	0.0013	0.0431	0.0482	< 0.0001^***^
TB (ref = No)					
Yes	0.0164	0.0114	-0.0059	0.0387	0.1502
Smoking (ref = No)					
Yes	0.0682	0.0098	0.0489	0.0874	< 0.0001^***^
Treatment type (ref = Others)					
Enalapril	-0.0139	0.0105	-0.0345	0.0067	0.1856
Nifedipine	-0.0354	0.0183	-0.0713	0.0005	0.0531
Enalapril + Nifedipine	-0.0879	0.0392	-0.1647	-0.011	0.0249^***^
Follow-up time	-0.0581	0.0059	-0.0697	-0.046	< 0.0001^***^
**Random effects**	**SD**				
Intercept ($\:{b}_{0i}$)	0.2171				
Visit time ($\:{b}_{1i}$)	0.0177				
Corr$\:\left({b}_{0i},{b}_{1i}\right)$	-0.488				
Residual ($\:{\epsilon\:}_{i}$)	0.2236				

## Discussion

In this study, we analyzed the longitudinal progression of pulse pressure (PP) among hypertensive patients and identified significant factors influencing its variability. PP, defined as the difference between systolic blood pressure (SBP) and diastolic blood pressure (DBP), reflects arterial stiffness and has been shown to predict cardiovascular risk independently. Our findings contribute to the growing body of evidence on the clinical relevance of PP as a critical biomarker in hypertension management. The main objective of this study was to identify factors that influence the longitudinal pulse pressure of hypertensive patients treated at Assosa General Hospital, Ethiopia, using linear mixed model analysis.

In the longitudinal data analysis, the pulse pressure (PP) measurements were first checked for normality using a Q-Q plot. The plots indicated a deviation from normality, necessitating some transformation. After applying a natural logarithm (ln) transformation to the PP, the mean response of the longitudinal lnPP was determined to be normal. The data were then analyzed using the transformed data, and the analysis was conducted using a random intercept and random slope model with an unstructured covariance structure, as it had smaller AIC and BIC values compared to the other random effects and covariance structures, respectively.

Our findings show that PP is positively associated with factors such as age, urban residence, family history of hypertension (FHHTN), diabetes mellitus (DM), chronic kidney disease (CKD), smoking status, and the stages of hypertension. These results align with evidence that increased large artery stiffness, as influenced by these risk factors, contributes to elevated PP [8]. This Framingham Heart Study highlighted that higher PP, especially in middle-aged and elderly individuals, is a marker of arterial stiffness and an independent predictor of coronary heart disease (CHD)​.

As we have seen in the individual profile plot from Fig. 1, the mean of the longitudinal PP was linearly decreasing with no systematic pattern over time. This indicates that the linearity assumption of the data was fulfilled. Then, the transformed data was analyzed using the linear mixed effects model by incorporating subject-specific variability.

The profile plot in our study revealed that PP decreased gradually over the follow-up time, likely due to the impact of antihypertensive treatment and lifestyle interventions. The gradual reduction in PP over the follow-up period, as shown in the profile plot, underscores the potential impact of targeted interventions and antihypertensive therapy. Treatments such as Enalapril and Nifedipine were negatively associated with PP, suggesting that effective management can attenuate arterial stiffness and reduce cardiovascular stress. This observation aligns with the work of [8], who emphasized the clinical utility of PP in predicting coronary heart disease risk; our results complement the findings from [6], which suggest that the pulsatile component of blood pressure is a more reliable marker of cardiovascular outcomes compared to peripheral blood pressure. As demonstrated in both our research and the referenced studies, interventions targeting the pulsatile components of blood pressure may hold promise in reducing cardiovascular risk.

The study revealed that the average PP increases with age. This result was consistent with another study [8, 15, 32–35]. In their finding, PP increases as age increases. This result was also consistent with [36]. Their findings indicated that women had lower pulse pressure levels than men during early adulthood, but these levels were higher in older age. Women experienced a more consistent and steeper increase in pulse pressure with age compared to men, who exhibited a more pronounced curvilinear rise in pulse pressure as they aged.

The apparent contradiction in our findings, where pulse pressure (PP) decreased gradually over the 24-month follow-up period but increased with age, can be explained by the differing time scales and factors influencing these trends. The short-term decrease in PP reflects the impact of antihypertensive treatments and lifestyle modifications, which are effective in reducing arterial stiffness and improving vascular health during the study period. In contrast, the positive association between PP and age represents the long-term physiological effects of aging, such as cumulative arterial stiffening and reduced vascular compliance. Additionally, older participants likely entered the study with higher baseline PP, and while treatment reduced PP across all participants, the age-related baseline differences remained evident. These findings highlight that PP dynamics are influenced by both modifiable factors, such as treatment and adherence, in the short term, and non-modifiable factors, such as aging, in the long term. Addressing these dynamics provides valuable insight into the importance of both immediate interventions and long-term cardiovascular management.

The average PP was found to evolve differently between patients from urban and rural areas. The average PP of urban patients was significantly higher as compared to rural patients. This result was consistent with [37, 38]. In their findings, urban patients have a higher risk of hypertension and greater difficulties in controlling their blood pressure compared to rural patients. Also, this result is consistent with another study [39]. Their findings indicate that although hypertension is common in both urban and rural Gambia, there is a higher prevalence of cardiovascular risk factors in urban areas. However, this result was contradicted by [40], which found that rural African Americans are at greater risk of poor diabetes and hypertension control. This discrepancy may be due to differences in demographics, socioeconomic status, healthcare access, and environmental factors such as stress and diet. Rural populations often have more active lifestyles and consume fewer processed foods, which can mitigate hypertension and elevated pulse pressure (PP) despite limited healthcare access. Additionally, variations in study design, population characteristics, and healthcare system organization may contribute to these contrasting findings, underscoring the need for further research.

The average PP of the patients with FHHTN was significantly higher as compared to the patients with no FHHTN. This result was consistent with [15, 41–44]. This result was also consistent with another study [45]. In their findings, a family history of hypertension, diabetes mellitus, and being overweight were associated with high blood pressure.

The average PP of Stage-I and Stage-II HTN patients was significantly higher as compared to Pre-stage HTN patients. This result was consistent with the study [42]. This result was also consistent with the study [46]. Their finding revealed that the management of blood pressure has improved among hypertensive adults, resulting in a higher percentage of individuals with blood pressure at optimal or prehypertension levels and a lower percentage in stages I and II hypertension.

The average pulse pressure (PP) of patients with diabetes mellitus (DM) was significantly higher compared to patients without DM. This result was consistent with studies [15, 16, 35, 45]. Their findings indicated that patients with diabetes mellitus were at risk of having higher blood pressure. This result was also consistent with study [47], which concluded that in type 2 diabetes, pulse pressure is positively associated with cardiovascular mortality.

The average PP of patients who had CKD was significantly higher as compared to patients without CKD. This result was consistent with another study [48, 49]. In their findings, elevated pulse pressure can negatively affect kidney health, potentially leading to a faster progression of chronic kidney disease. The average PP of smoker patients was significantly higher as compared to non-smokers. This result was consistent with [35, 50]. In their findings, hypertensive smokers were more likely to develop severe forms of hypertension and had higher recorded blood pressure measurements.

The findings of this study suggest that as the patients’ follow-up time increases, their average pulse pressure decreases, indicating better control of their pressure by following their treatment (Enalapril + Nifedipine). This result was consistent with another study [51]. In their findings, as follow-up time increased, the patient’s blood pressure decreased slowly. This result was also consistent with another study [52]. Their findings, Enalapril and Nifedipine are both effective antihypertensive drugs, and in some hypertensive patients, their effects appear to be synergistic.

### Limitation

This study contributes to the limited body of research focusing on pulse pressure (PP) as a biomarker for hypertension (HTN) and associated cardiovascular diseases (CVD). While previous studies have primarily measured systolic blood pressure (SBP) and diastolic blood pressure (DBP), there is a scarcity of longitudinal investigations specifically analyzing PP. Existing PP-focused studies are often cross-sectional, underscoring the need for further research to explore PP as a critical biomarker in HTN and related CVD.

However, this study has several limitations. The retrospective design may introduce potential biases, such as inaccuracies or inconsistencies in medical record documentation, which could affect the reliability of the data. Additionally, the observational nature of the study limits the ability to infer causal relationships between PP and its associated factors. Future prospective studies with rigorous data collection methods are recommended to validate these findings and further explore the role of PP in cardiovascular health.

## Conclusion

This study was a retrospective analysis based on 325 hypertensive patients undergoing follow-up for antihypertensive treatments at AGH. The gradual reduction in PP over the follow-up time, as shown in the profile plot, underscores the potential impact of targeted interventions and antihypertensive therapy. Age, residence, family history of hypertension (FHHTN), diabetes mellitus (DM), chronic kidney disease (CKD), smoking status, and stages of HTN were positively associated with PP, whereas treatment type and follow-up time were negatively associated with PP. As a recommendation, healthcare providers should prioritize addressing the modifiable risk factors mentioned above to help mitigate the progression of blood pressure, specifically pulse pressure (PP), in hypertensive patients.

## Electronic supplementary material

Below is the link to the electronic supplementary material.

Supplementary Material 1

Supplementary Material 2

## Data Availability

The datasets generated and/or analyzed during the current study are not publicly available due to ethical concerns, confidentiality agreements, or legal restrictions. However, the data can be obtained by contacting the corresponding author of the study and making a reasonable request for access to the data.

## Abbreviations

WHO: World Health Organization
AGH: Assosa General Hospital
SE: Standard Error
DM: Diabetes Mellitus
CKD: Chronic kidney disease
HTN: Hypertension
CVD: Cardiovascular disease
PP: Pulse pressure
lnPP: Natural logarithm of Pulse Pressure
DBP: Diastolic blood pressure
SBP: Systolic blood pressure
TB: Tuberculosis
AIC: Akaike Information Criterion
BIC: Bayesian Information Criterion
