# Supplementary figures and images for: Determinant factors of the longitudinal pulse pressure among hypertensive patients treated at Assosa general hospital, Western Ethiopia
Source: BMC Cardiovasc Disord. 2024 Dec 19;24:715. doi: 10.1186/s12872-024-04389-7 (PMC11660837; doi:10.1186/s12872-024-04389-7)

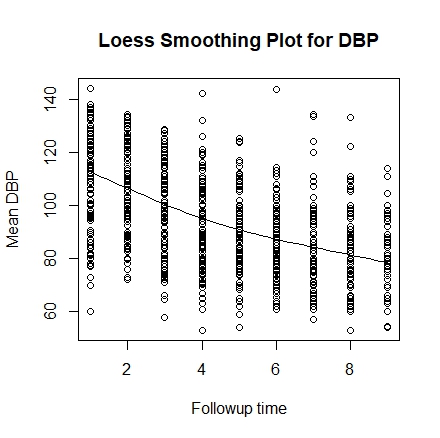


**SuppFigure 1**: Loess Smoothing Plot for Diastolic Blood Pressure

Supplement: Supplementary file 1 — Supplementary Material 1 [file 12872_2024_4389_MOESM1_ESM.docx]

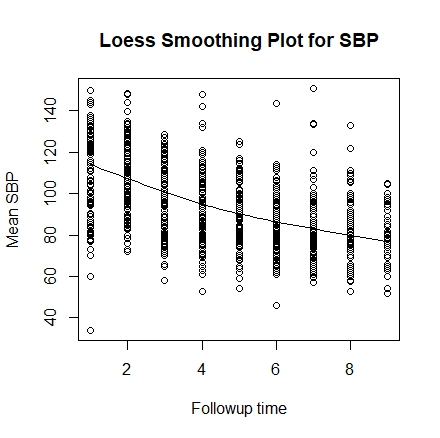


**SuppFigure 2**: Loess Smoothing Plot for Systolic Blood Pressure

Supplement: Supplementary file 2 — Supplementary Material 2 [file 12872_2024_4389_MOESM2_ESM.docx]
